# Prognosticating the outcome of intensive care in older patients—a narrative review

**DOI:** 10.1186/s13613-024-01330-1

**Published:** 2024-06-22

**Authors:** Michael Beil, Rui Moreno, Jakub Fronczek, Yuri Kogan, Rui Paulo Jorge Moreno, Hans Flaatten, Bertrand Guidet, Dylan de Lange, Susannah Leaver, Akiva Nachshon, Peter Vernon van Heerden, Leo Joskowicz, Sigal Sviri, Christian Jung, Wojciech Szczeklik

**Affiliations:** 1 Department of Medical Intensive Care, Hadassah Medical Center and Faculty of Medicine, Hebrew University of Jerusalem, Jerusalem, Israel; 2 Unidade Local de Saúde São José, Hospital de São José, Lisbon, Portugal; 3 Centro Clínico Académico de Lisboa, Lisbon, Portugal; 4 Faculdade de Ciências da Saúde, Universidade da Beira Interior, Covilhã, Portugal; 5 Center for Intensive Care and Perioperative Medicine, Jagiellonian University Medical College, Krakow, Poland; 6 Institute for Medical Biomathematics, Bene Ataroth, Israel; 7 Imperial College Business School, London, UK; 8 Department of Research and Development, Haukeland University Hospital, Bergen, Norway; 9 INSERM, Institut Pierre Louis d’Epidémiologie Et de Santé Publique, AP-HP, Hôpital Saint Antoine, Sorbonne Université, Service MIR, Paris, France; 10 Department of Intensive Care Medicine, University Medical Center, University Utrecht, Utrecht, The Netherlands; 11 General Intensive Care, St George’s University Hospitals NHS Foundation Trust, London, UK; 12 General Intensive Care Unit, Department of Anaesthesiology, Critical Care and Pain Medicine, Faculty of Medicine, Hebrew University and, Hadassah University Medical Center, Jerusalem, Israel; 13 School of Computer Science and Engineering and Center for Computational Medicine, The Hebrew University of Jerusalem, Jerusalem, Israel; 14 Department of Cardiology, Pulmonology and Vascular Medicine, Faculty of Medicine, Heinrich-Heine-University, University Duesseldorf, Moorenstraße 5, 40225 Düsseldorf, Germany

**Keywords:** Intensive care, Critical care, Prediction, Very old patients

## Abstract

Prognosis determines major decisions regarding treatment for critically ill patients. Statistical models have been developed to predict the probability of survival and other outcomes of intensive care. Although they were trained on the characteristics of large patient cohorts, they often do not represent very old patients (age ≥ 80 years) appropriately. Moreover, the heterogeneity within this particular group impairs the utility of statistical predictions for informing decision-making in very old individuals. In addition to these methodological problems, the diversity of cultural attitudes, available resources as well as variations of legal and professional norms limit the generalisability of prediction models, especially in patients with complex multi-morbidity and pre-existing functional impairments. Thus, current approaches to prognosticating outcomes in very old patients are imperfect and can generate substantial uncertainty about optimal trajectories of critical care in the individual. This article presents the state of the art and new approaches to predicting outcomes of intensive care for these patients. Special emphasis has been given to the integration of predictions into the decision-making for individual patients. This requires quantification of prognostic uncertainty and a careful alignment of decisions with the preferences of patients, who might prioritise functional outcomes over survival. Since the performance of outcome predictions for the individual patient may improve over time, time-limited trials in intensive care may be an appropriate way to increase the confidence in decisions about life-sustaining treatment.

## Introduction

Identifying the patients with critical illnesses who will benefit from intensive care remains a challenge. Human judgement is imperfect [1, 2] and mistakes are made-either by withholding or withdrawing intensive care in patients who might benefit from life-sustaining treatment (LST) or by exposing others to non-beneficial interventions. This problem is documented by the survival rates in patients with conditions deemed as unsurvivable and the variability of decisions to withdraw LST, resulting in ethical controversies [3–5]. Statistical models have been developed with empirical datasets to assist clinicians with prognostication to varying degrees of success [6]. These shortcomings are reflected by the growing interest in time-limited trials in the intensive care unit (ICU) to manage prognostic uncertainty in the individual patient [7].

Very old patients (chronological age ≥ 80 years) pose a particularly challenging problem for prognostication [8–11]. The age-related decline of physiological and cognitive reserves proceeds at different rates [12]. Its effect on the presentation and severity of acute disorders is significant but hard to measure [13]. Chronic co-morbidities and multi-morbidity, which are highly prevalent in old age, and the reduced resilience to acute stress (frailty) may influence the trajectory of critical conditions in various ways, before admission, during the stay in ICU and after discharge [14, 15]. The resulting heterogeneity within the group of very old patients compromises the performance of the current armamentarium for prognosticating the outcomes of intensive care [16, 17].

Whereas surviving a critical illness is the most crucial goal in younger patients, many very old patients value functional abilities and quality of life after discharge more than physical survival. Thus, younger and older individuals’ views and expectations about intensive care may differ. Since the use of advanced directives is limited in many countries and the performance of tools for prognostications in these domains remains suboptimal [18], the ultimate prediction of ICU outcomes and the alignment of the subsequent decisions about LST with the patient’s preferences are mostly left to the discretion of healthcare professionals or surrogate decision-makers. Their cultural and individual preferences can lead to substantial biases and misalignments of decisions regarding the patients' wishes [19–22]. As a result, societal norms may become self-fulfilling prophecies for ICU outcomes, which might be perpetuated in future prediction models trained on today’s data.

Poor prognostication can eventually lead to inappropriate care for the individual patient and inefficient utilisation of ICU assets [23–25]. Substantial resources are being invested to improve predictive modelling and, hence, decision-making about the level of LST that is best for the individual [26–28]. This article will discuss both classical and new approaches addressing these challenges.

## Predictive modelling

The classical approach to building instruments for predicting (prognosticating) future events or states (outcome), such as survival or functional independence at a specific time, is fitting regression models to distributions of patient characteristics before these events, such as the severity of the critical condition, and the outcome of interest. Logistic regression is used when the outcome is dichotomous, such as survival vs non-survival.

There are only a small number of studies which focused on predictive modelling in ICU patients aged 80 years or older. Screening of Pubmed (www.pubmed.gov) for articles with a special emphasis on this particular cohort using the query “(older[ti] OR “very old”[ti] OR “oldest old”[ti] OR elderly[ti]) AND patient* AND (“intensive care” OR “critical care”) AND (outcome* OR survival OR mortality) AND (prognos* OR predict*) AND model*” identified five original studies published in the past 10 years [29–33], after the exclusion of studies using databases and those involving disease-specific sub-groups or published by our own group.

**Fig. 1:**
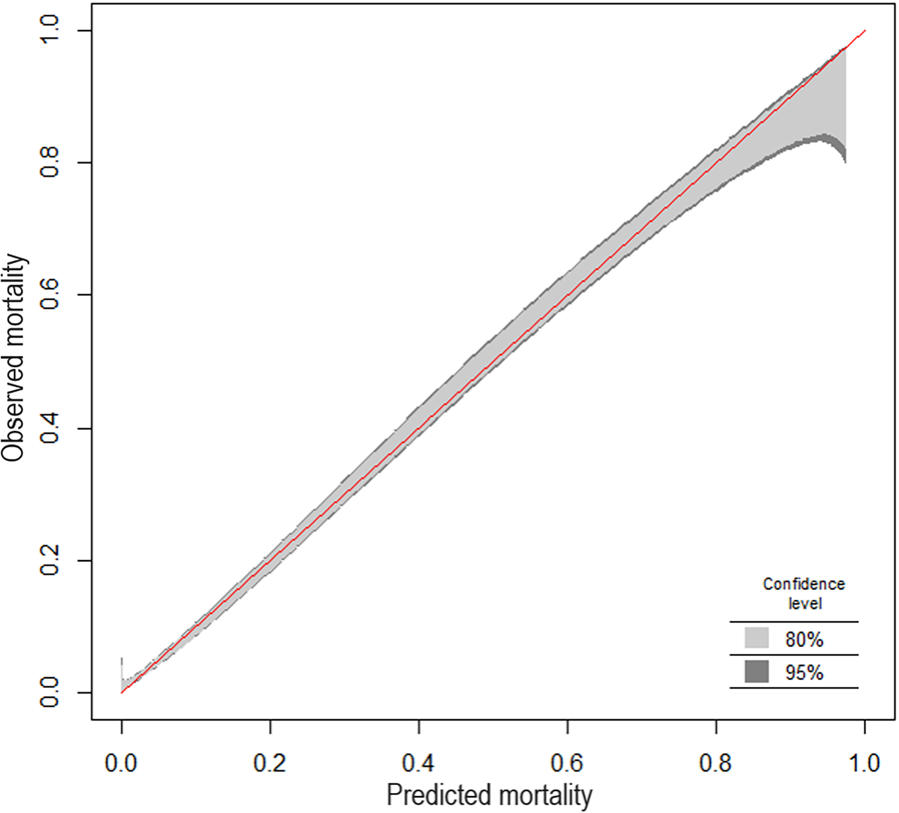
Calibration belt for 16 784 patients from the SAPS 3 database [34, 35] describing the relationship between predicted and observed hospital mortality, which is depicted as the fraction of non-survivors. The plot was generated using the R package givitiR [36]. The overlay between the identity function (red line) and the calibration belt with narrow confidence intervals (shaded areas-see inset) indicates a good agreement between model predictions based on the SAPS 3 score and observed outcomes for most of the range of hospital mortality

**Fig. 2:**
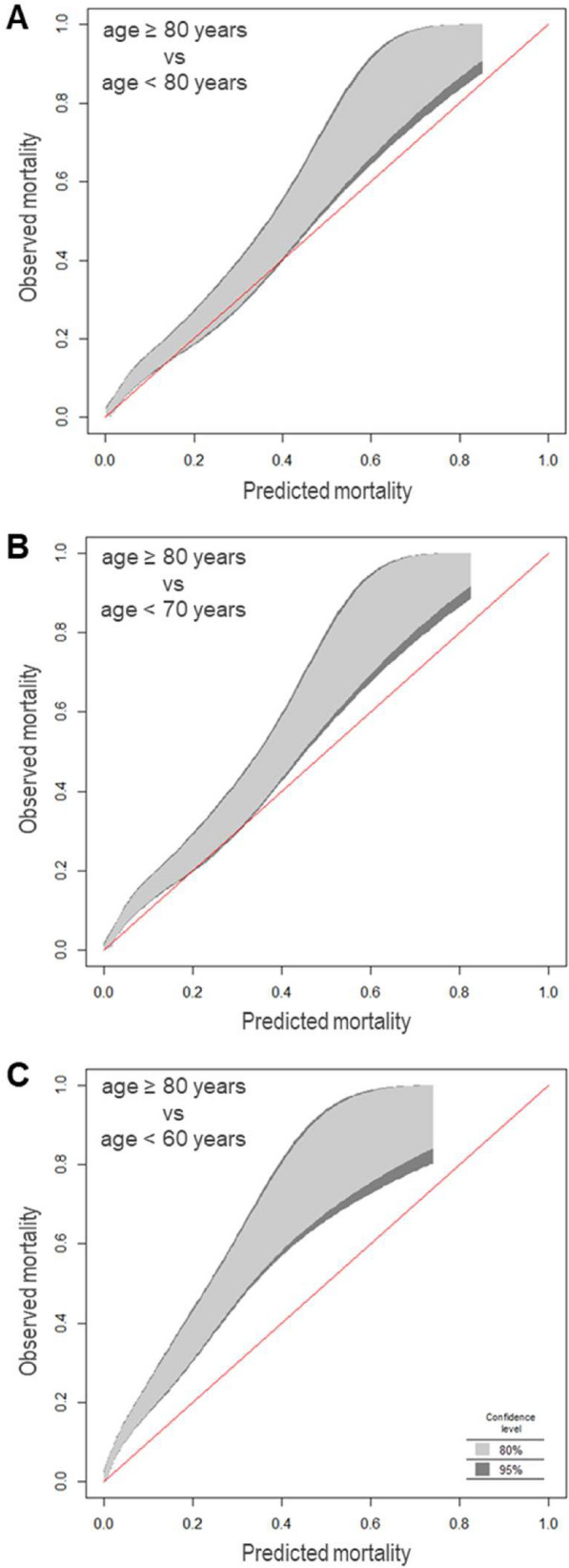
Illustration of the impact of chronological age on the calibration of prediction models. ICU mortality is depicted as the fraction of non-survivors and was predicted based on SOFA scores in patients from the eICU database [37]. The plots were generated using the R package givitiR [36]. The shaded regions depict calibration belts for various confidence levels (see inset). Panels **A**–**C** show the calibration characteristics for models trained on patients younger than the indicated threshold [**A**: ≤ 60 years (n = 7 928), **B**: ≤ 70 years (n = 13 612), **C**: ≤ 80 years (n = 19 237)] and applied to patients aged 80 years or older (n = 5 252). The extent and shape of the calibration belts’ deviation from perfect calibration (red line) depend on the age gap between the training and deployment samples

The performance of predictive models is assessed by quantifying calibration and discrimination in different cohorts. Calibration is the measure of the statistical agreement of model predictions with observed outcomes. For example, if 80 out of 100 patients survive in a particular group, a perfectly calibrated model generates a mortality prediction of 80% for that group. Figure 1 demonstrates this relationship as a calibration plot for the hospital outcome cohort of the Simplified Acute Physiology Score (SAPS) 3 study which included 16 784 patients from 303 ICUs around the globe [34, 35]. Figure 2 illustrates the effect of age on calibration in a simple mortality model using the severity of organ dysfunction to predict ICU survival. A cohort of 24 489 adult patients with available sequential organ failure assessment (SOFA) scores was extracted from the eICU database [37]. ICU mortality was calculated for each SOFA score value in the younger training cohort and compared to that observed in a cohort of 5252 patients aged 80 years or older using the software R (version 4.1.1, www.r-project.org). The models trained on younger cohorts underestimate mortality for very old patients across parts or the whole of the spectrum of predictions (Fig. 2). Since there is no gold standard to evaluate calibration [38], the degree of miscalibration deemed acceptable is, to some degree, subjective and context-dependent. Clinicians should check whether the prediction model is well calibrated for the particular case-mix in their ICU.

**Fig. 3:**
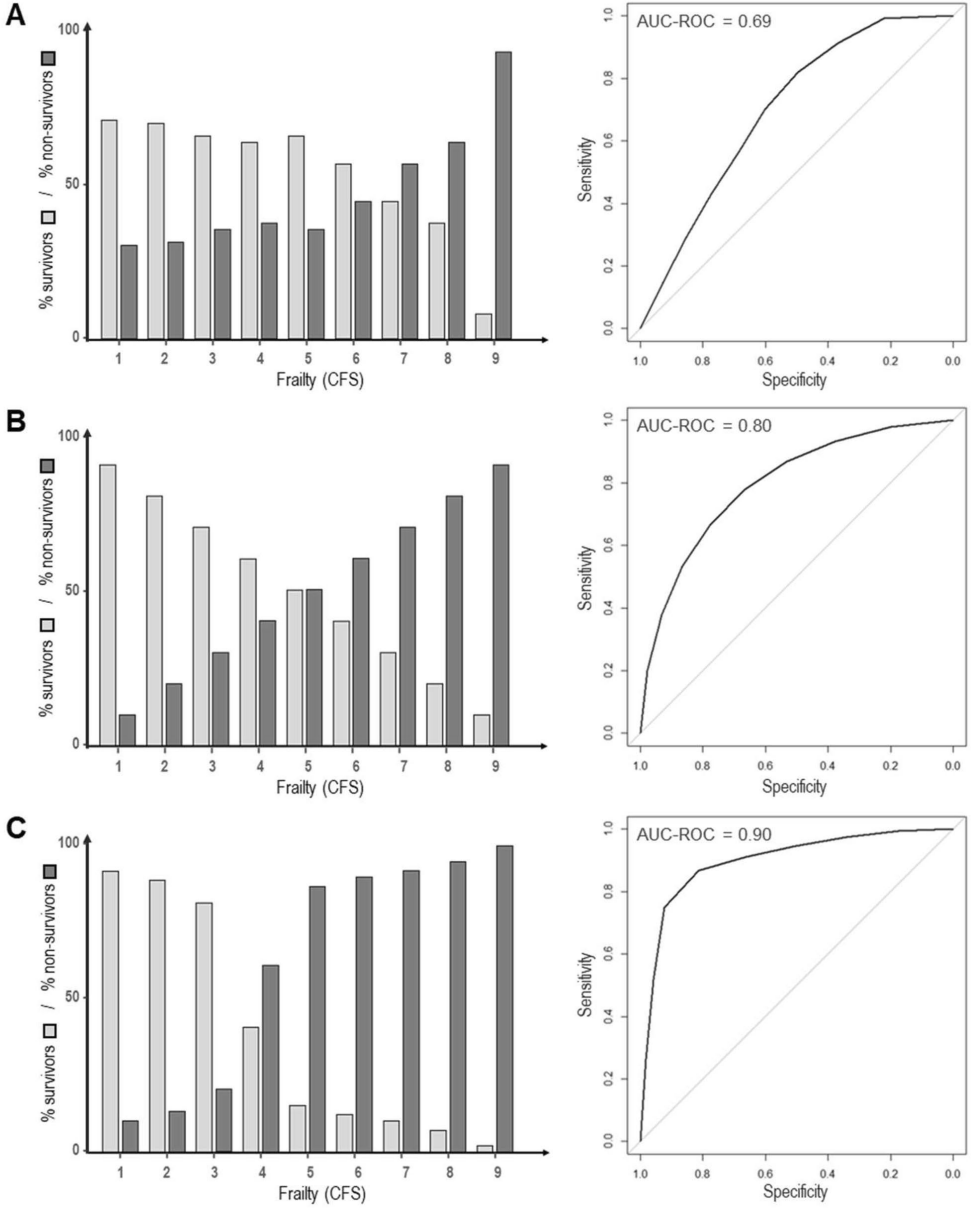
Illustration of discrimination of prediction models for simulated distributions of survival for various degrees of frailty, which was measured with the Clinical Frailty Scale (CFS) [39]. Percentages of ICU survivors and non-survivors per CFS category are depicted by light and dark bars, respectively, on the left side. The corresponding receiver operating characteristic (ROC) curves for survival predictions and the area under these curves (AUC-ROC) were obtained using the R package pROC [40] and are shown on the right side. Panel A depicts distributions of ICU survivors and non-survivors using information from the VIP2 study about the percentages in each CFS category [41]. Overall discrimination between the two outcomes (AUC-ROC = 0.69) is sub-optimal. Panel B shows a hypothetical scenario with a linear relationship between frailty (CFS) and ICU mortality. Despite the good AUC-ROC of 0.8, there is maximum uncertainty, i.e. a 50% chance of survival, for patients with CFS 5 (mild frailty). Panel C presents another hypothetical scenario resulting in an AUC-ROC of 0.9 which indicates a very good discrimination for the prediction model. However, the overlap at CFS 4 (very mild frailty) leads to substantial prognostic uncertainty for the patients in this particular frailty category

Discrimination describes the model’s ability to differentiate between groups of patients with distinct outcomes. This concept is crucial for assessing the merit of models in clinical practice, when outcomes are fundamentally different, such as survival and non-survival, and decisions are irreversible. Overall discrimination can be measured using the area under the curve of the receiver operating characteristic (AUC-ROC), which relates the sensitivity and specificity of a dichotomous prediction model. It ranges from 0.5, indicating discrimination no better than the random results after tossing a coin, to 1.0 for perfect discrimination. An AUC-ROC value of 0.9 implies that in 90% of the cases, the model correctly ranks a patient with a particular event, such as survival, higher than the one without. Figure 3 depicts AUC-ROCs for simulated distributions of ICU survivors and non-survivors having variable degrees of pre-existing frailty [39]. The values for these AUC-ROCs range from 0.69, which represents suboptimal discrimination, to 0.9 which indicates very good discrimination. Despite good AUC-ROC values though, there can be a marked overlap of the distributions as illustrated by the simulated examples in Fig. 3. This complicates prognostication for those patients with a degree of frailty that is contained within the overlap. Thus, the discrimination of a predictive model should also be verified for the range of values that is relevant to a particular patient group.

The generalisability of prediction models refers to their performance in patient groups or healthcare settings other than the one used for training. Variations of patient biographies according to geography and era as well as differences in cultural norms and practice patterns affect care processes and outcomes [4, 42–44], especially in very old patients with complex conditions. Thus, the generalisability of many prediction models is limited [45, 46] and the performance of these models was shown to degrade when deployed in different healthcare settings or during different periods in time [47, 48].

## Disease severity scores

**Fig. 4:**
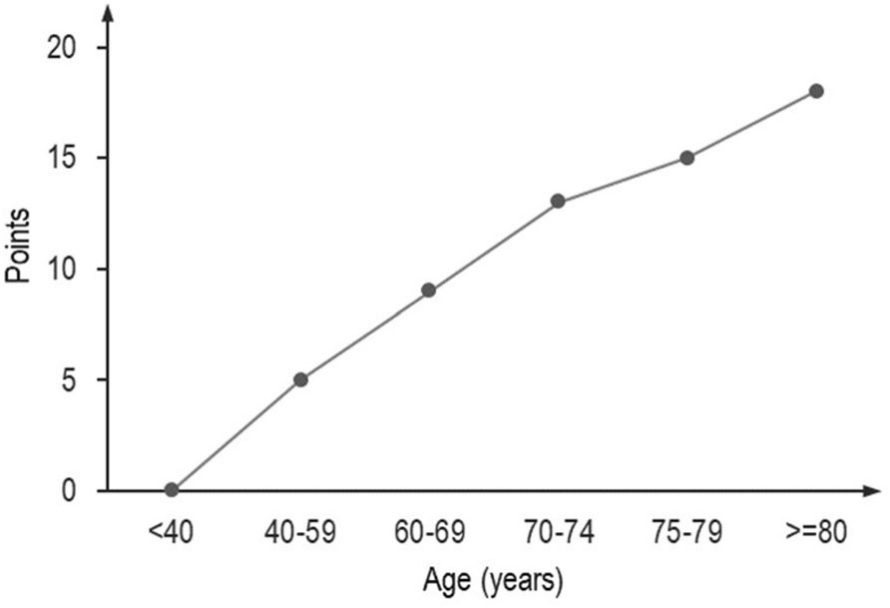
Impact of chronological age on the SAPS 3 score. The plot shows the points added to the score for patients in specific age categories. Patients aged 70 years or older get more points for their chronological age than younger patients would receive for a diagnosis of cirrhosis or cancer, which would add 8 or 11 points, respectively

Disease severity scores, such as the Acute Physiology and Chronic Health Evaluation (APACHE) score or the Simplified Acute Physiology Score (SAPS), were developed to predict survival at discharge from the hospital. The underlying models are based on three types of variables: (1) chronological age and chronic health status including co-morbidities, (2) circumstances of ICU admission, e.g. elective vs acute, and admission diagnosis and (3) markers of organ dysfunction observed at admission to the ICU or within 24 h thereafter. A significant increase in the risk of death in patients older than 40 years in comparison to younger cohorts, even when controlling for other demographic and physiological variables, was observed while developing disease severity scores [34]. This is reflected by the points assigned to different age groups for the SAPS 3 score (Fig. 4).

In addition to scoring disease severity with data obtained at a single point in time, this method can also be applied in a dynamic way that includes the physiological response to interventions assessed at regular intervals in the ICU [49]. This approach has demonstrated the potential to outperform conventional scores with respect to the discrimination of survivors vs non-survivors [50]. Daily recordings of the SOFA score [51] allow the computation of time-dependent measures, such as the delta SOFA score, which quantifies the evolution of the critical illness in response to interventions and can improve outcome predictions [52].

Chronic conditions and impairments, which determine the health status in most very old patients, are established predictors of ICU survival [53, 54]. They were shown to have an increasing statistical impact on in-hospital mortality the longer patients are in intensive care [55] and a profound influence on survival at 6 months in very old patients [29]. However, frequent geriatric conditions, such as neurocognitive disorders, are still not taken into account by the classical disease severity scores. Moreover, the chronic health status in very old patients should ideally be evaluated by a comprehensive geriatric assessment to capture the distinct risk profile in the individual [56, 57]. This includes additional dimensions, such as socio-economic features, which were shown to affect outcomes of critical conditions in this group [58]. Due to the time constraints in managing critically ill patients, this approach may only be feasible in the workup for planned ICU admissions in the context of elective surgery. However, it would be important to adapt fundamental aspects of a geriatric assessment to emergency care in the ICU as recently outlined by Jacobs et al. [59].

The overall contribution of chronic conditions and chronological age to the prediction of survival with disease severity scores increased in the past decades [35]. In contrast, the explanatory power of markers of acute organ dysfunction is decreasing. This can be related to the progress of intensive care in managing acute organ dysfunction. Yet, the ability to deal with the consequences of chronic conditions and impairments which progress with ageing and declining physiological reserves, including reduced cardiovascular fitness, immunosenescence and the impact of degenerative diseases, is still very limited. Importantly, the performance of disease severity scores is affected by ongoing demographic shifts, with today's very old patients not being adequately represented in historical samples for training models, and the fact that some conditions, which were imminently fatal in the past, are now managed as chronic conditions over an extended period. On this background, the discriminatory performance of the APACHE IV and SAPS II scores was found to be worse for very old patients [16, 60]. In addition to the above issues, the APACHE score requires choosing a principal admission diagnosis, which can lead to ambiguity in older patients with complex multimorbidity and further impair the model's performance.

There is an ongoing discussion about replacing chronologic age with biological age to enhance the predictive accuracy of disease severity scores in very old patients [61]. However, this requires a robust definition of biological age. Even if such a definition would focus on frailty as a marker of age-related vulnerability to stress [13], a comprehensive assessment of the individual patient beyound a simple screening might be necessary to produce a reliable and meaningful impact on the performance of prediction models. Similar considerations may apply to other geriatric conditions, notably multimorbidity [62]. Some studies have already combined measures of acute organ dysfunction with frailty screening and showed promising results for the predictive discrimination of short-term survival in sub-groups of very old ICU patients [63, 64]. In particular, phenotyping of patients 80 years or older based on a combination of SOFA scores and the clinical frailty scale (CFS) at ICU admission resulted in seven distinct sub-groups with different mortality at 30 days, ranging from 3 to 57%. Importantly, the phenotype representing mostly nonagenarians with a median CFS level of 4 (very mild frailty) and a median SOFA score of 4 had a mortality of only 7% at 30 days after ICU admission, in contrast to less old patients with more advanced frailty and similar SOFA scores [64].

## Prediction of functional outcome and quality of life

Survival is an essential milestone of intensive care. However, recovering the pre-existing level of independence in daily life and self-perceived quality of life are other major goals [65]. They are especially important for very old ICU survivors with frailty, which confers an increased vulnerability to stress with potentially long-term sequelae for functional abilities. Few studies attempted to predict functional outcomes or quality of life for old individuals, since such investigations usually involve comprehensive assessments by geriatric teams [57]. Ferrante et al. [66] used age, frailty, pre-existing disabilities, depressive symptoms, previous hospitalisations and hospital length of stay to predict persistent functional impairment in the year after discharge from the ICU, though with only moderate discrimination (AUC-ROC 0.71). In another study, severe limitations of health-related quality of life were found in half of the patients with COVID-19, who were 70 years and older, three months after ICU admission and to be associated with pre-existing frailty [67]. However, the generalisability of this particular finding might be limited by the admission biases caused by the constraints on ICU resources during the COVID-19 pandemic [68].

According to an international expert consensus, predicting functional outcomes and quality of life is generally compromised by obstacles to quantifying these measures robustly [69]. Future instruments to assess the quality of life in a very old person should consider the specific expectations and priorities in this age group, which may differ significantly from those in younger cohorts. There also is a broad spectrum of contextual confounders, ranging from individual preferences of decision-makers to access to healthcare services, which may influence these functional outcomes [21]. Moreover, patients' attitudes are known to fluctuate over time [70]. Therefore, the performance of current methods for predicting outcomes in the above domains does not appear to be robust enough for determining major decisions about LST.

## New machine learning technologies

The number of patient characteristics found to correlate with outcomes of intensive care continues to rise, notably when considering the large set of features which can be provided by geriatric assessments [56] or dynamic predictions in the ICU [49]. The resulting expansion of patients’ datasets in size and dimensionality constitutes a formidable challenge for predictive modelling [71]. New technologies in machine learning (ML) were designed to process large datasets to identify relationships between multiple variables describing patients, context and care processes. Until recently, most of these technologies were based on scaling-up classical methodologies, such as logistic regression in artificial neural networks or decision trees in random forests.

The quality and granularity of data for training remains pivotal for the performance of models, even when using 'big data' [26]. This concerns noise in recordings of physiological and biochemical variables, incorrect documentation of interventions and the omission of decisions about LST [20]. Moreover, cultural and professional norms, such as local policies of limiting LST, influence outcome and are implicitly integrated in historical datasets. They may affect the generalisability of prediction models if their association with outcome remains undetected during the development and deployment of these models.

New and more sophisticated types of neural networks (transformers) were developed by enhancing the interconnections between their sub-units to detect more complex patterns in data [72, 73]. This technology is evolving rapidly, and we would like to refer the interested reader to the latest literature in this domain. Foundation models, often known as large language models, are based on the transformer architecture and represent today's frontier in ML technologies and artificial intelligence [74]. Factuality and adversarial safety of these models have been a problem but can be improved by adding verification with data from trusted sources [75–77]. The versatility of foundation models has been illustrated by the performance of models processing a broad spectrum of knowledge in different domains and, importantly, showing reasoning capabilities [78]. These capabilities appear to be an emergent feature of very large models and may eventually enhance the generalisability of the embedded information [79]. Smaller models can be effectively trained by focussing on domain-specific data, such as clinical patient notes [28]. This data source also contains information about professional norms and care processes, which is usually not included in classical prediction models. A large hospital system has applied this new technology to generate outcome predictions for an unselected cohort of almost 400 000 patients admitted to hospital during the period 2011 to 2020 [28]. Although discrimination for in-hospital mortality was excellent (AUC-ROC 0.95), it remains to be seen if this performance level can be replicated with respect to the characteristics and outcomes in very old patients.

Although numerous ML models have been developed for outcome predictions in intensive care for unselected patient populations or disease-specific sub-groups [80], there is a scarcity of ML models designed for prognostication in very old patients using the specific set of geriatric characteristics in this cohort [57]. In the one study published in this field, an augmented version of decision tree analysis was employed to provide survival predictions based on six categories of variables, including demographic and acute patient characteristics as well as features of frailty and multi-morbidity. The resulting model showed only a slightly better discrimination than classical disease severity scores, such as SAPS II and APACHE IV, for patients older than 65 years [17]. However, sub-group analyses revealed a decreased discrimination for in-hospital mortality in patients older than 80 years, which confirms findings in previous studies using conventional methods [16].

We have previously discussed the benefits and pitfalls of ML technologies and the related uncertainties about predictions from an ethics point of view [81]. The recent advent of large foundation models has only heightened the concerns underlined in that article. This especially applies to the selection of unbiased datasets for training, e.g. by assessing the incidence of decisions to limit LST which is expected to be very variable [25], and the framework for an oversight of algorithms to detect inappropriate outputs [75]. Moreover, the importance of transparency and explainability of ML models in defining their utility in clinical practice remains a matter of debate [82]. Methods which estimate the effect of a specific patient characteristic on outcome predictions can provide a solution for the issue of transparency [83]. Yet, the size of the effect of a variable on the model's output does not explain the parameter's specific biological role, which would be useful for causal reasoning.

## From outcome predictions to decisions-the challenge of uncertainty

Current prediction models are designed to generate a probability for a specific outcome derived from the statistical properties of training cohorts. The resulting number, however, can only be integrated into the decision-making for individual patients with additional considerations. First, decision-makers have to verify that a prediction model is applicable within the specific context of the case since variations of care processes between healthcare settings may substantially alter the calibration and discrimination of models [47]. In particular, recommendations for specific cut-offs with respect to major decisions about LST should be scrutinised for unwanted consequences [84]. Second, even if the patient and context variables matched the training cohort, the probabilistic nature of model predictions requires dichotomisation of the model output into categories of actionable decisions when, for example, contemplating withholding or withdrawing LST in the individual patient. This process necessitates an understanding of the uncertainty regarding both the predictions of the model in general and the specific risk given for an individual patient [85, 86]. Uncertainty can be quantified using the concept of entropy [87] and monitored over time to understand how the confidence in outcome predictions evolves during critical care [88]. This might be particularly relevant to the cohort of older patients due to their inter-individual heterogeneity, which results in a high degree of uncertainty in statistical predictions when provided only at a single point in time.

Prognostication is a prelude to decision-making in intensive care. Decisions should be based on an objective description of the critical condition to be fair and robust. They should also be aligned with the patient's individual preferences which is especially relevant to individuals with complex co-morbidities [89, 90]. However, discussing predictions about likely outcomes with patients or surrogates can be challenging. This step benefits from suitable communication skills to translate numerical data, i.e. probabilities, into information that can be processed by lay people during decision-making. The finding that better numeracy in patients is associated with better outcome [91] indicates the need to further improve these skills among healthcare professionals.

The impact of predictive uncertainty on the decision-making in clinical practice has been acknowledged by clinicians as well as professional institutions [9, 92]. A time-limited trial (TLT) in the ICU can be a suitable intervention if the uncertainty about the predicted outcome is uncomfortably high. A TLT is a collaborative plan among clinicians and the patient or surrogate decision makers to use LST for a defined duration, after which the patient’s response to this treatment informs the decision to continue or withdraw LST [7]. On this background, a TLT provides the opportunity to gather additional information, notably about the patient's response to interventions [93], which can enhance the accuracy of prognostication [50]. However, a TLT may also result in increased uncertainty about survival, e.g. when new data obtained after stabilisation of organ function makes the predicted mortality drop from 80 to 50%, which represents the peak of uncertainty. These situations require a careful and comprehensive assessment as well as discussions with other stakeholders about individual goals of care and, thereby, the individual objective for prognostication [94]. Especially in very old patients, the predicted benefit of intensive care has to be weighed against its physiological and psychological burden in the context of an enhanced vulnerability to stress (frailty). Shared decision-making [89] may also include reflections on the impact of a potentially suboptimal outcome on the wellbeing of caregivers, when assessed from the patient's perspective.

## Discussion

The prediction of outcomes for the individual patient determines major decisions along the pathway of critical care [11]. This concerns decisions about admission to the ICU as well as those about continuation, escalation or withdrawal of LST in the ICU. Whereas prognostication at the point of a potential ICU admission is usually based on a fixed amount of information and made under time constraints, decision-making in the ICU can benefit from flexibility in the extent of time available for collecting more data and improve prognostication for the individual patient. Considering time as an additional dimension in predictive modelling, however, requires more sophisticated methods than those currently used in clinical practice.

When statistical models are employed for the purpose of prognostication, particular attention needs to be paid to the calibration and discrimination of these models in a specific healthcare setting. These factors are influenced by the generalisability of a model's underlying assumptions and contextual parameters, such as cultural norms for limiting LST or the availability of early rehabilitation for ICU patients [59]. Failure to acknowledge these issues may lead to faulty predictions and inappropriate care.

Additional assessments and reflections are necessary to integrate model-based predictions into robust decision-making about critical care. Reflections about prognostic uncertainty should go beyond a simple acknowledgement [9] and extend to quantification [85, 95]. Understanding the degree of the uncertainty of predictions is particularly relevant to dichotomous and irreversible decisions about LST. However, it remains to be determined what specific degree of uncertainty might be acceptable when translating model predictions into actions [92]. Additional steps might be necessary to manage situations of enhanced uncertainty. These include obtaining second opinions from experts outside the ICU team, such as from geriatricians, and engaging in a robust communication with patients or surrogate decision-makers.

The problem of the uncertainty of initial prognostic assessments can be mitigated by adding information about the patient's response to treatment over time [7]. This approach is of particular value for the cohort of very old patients with a high prevalence of various degrees of frailty, which confers an increased but poorly predictable vulnerability to the stress caused by critical conditions. Analysing time-dependent (dynamic) scores for organ failure and disease severity is a promising approach to enhance predictive accuracy. As data acquisition and processing are becoming fully automated, dynamic methods for prognostication may be developed into efficient tools for decision support. However, the burden of care as perceived by the individual patient, which is influenced by age-related conditions, has to be integrated into decisions about LST. Since there are no robust instruments for quantifying symptoms in this domain, the decision-making still requires a prudent judgment by the ICU team.

## Conclusions

Current methods for predicting outcomes of intensive care are imperfect and require careful implementation, especially for very old patients with complex chronic health status and variable personal expectations. The additional consideration of geriatric and time-dependent patient characteristics and a better generalisability of prediction models can further refine prognostication to support individualised decisions about LST in this patient cohort.

From our point of view, adjustments of the methods for prognostic modelling and their careful integration into the decision-making processes in the ICU may provide the opportunity for actionable prognostication in clinical practice (Table 1). The next generations of intensivists will have to acquire advanced knowledge about probabilistic reasoning to better understand the strengths and limitations of these technologies [96]. However, regardless of the refinement of data science technologies, the predictability of future events for the individual patient will remain limited due to the probabilistic nature of these methods. Therefore, conversations about prognostic uncertainty with patients and families are a crucial component of decision-making before admission to the ICU or in the ICU [94]. The variable preferences and wishes of stakeholders have to be balanced with predicted outcomes and their uncertainty when making decisions about LST [97]. In these situations, the trade of decision-making still remains an art.

**Table 1:** Problems of integrating prognostic modelling into decision-making processes in the ICU

Problem	Solutions
Generalisability of predictive models	Add variables describing specific legal, cultural and professional norms as well as available resources, which influence care processes, to prediction models
Prediction of functional outcome and quality of life in very old patients	Suitable training samples with respect to chronic conditions (e.g. frailty, multimorbidity) and impairments (e.g., assisted living) typical for very old age
Interpretability of model predictions	Incorporate explainability metrics
Alignment of data-informed decisions with patient's preferences	Document patient’s goals and expectations, share decision-making
Uncertainty of outcome predictions for individual patients	Time-limited trials with sequentially updated predictions, shared decision-making integrating the burden of treatment

## Data Availability

Not applicable.

## Abbreviations

APACHE: Acute physiology and chronic health evaluation (score)
AUC-ROC: Area under the curve of the receiver operating characteristic
CFS: Clinical frailty scale
ICU: Intensive care unit
LST: Life-sustaining treatment
ML: Machine learning
SAPS: Simplified acute physiology score
SOFA: Sequential organ failure assessment (score)
TLT: Time-limited trial
